# Exploring Current Evidence on the Past, the Present, and the Future of the Heart Team: A Narrative Review

**DOI:** 10.1155/2020/9241081

**Published:** 2020-01-04

**Authors:** Alexandru Burlacu, Adrian Covic, Mircea Cinteza, Paula Madalina Lupu, Radu Deac, Grigore Tinica

**Affiliations:** ^1^Department of Interventional Cardiology, Cardiovascular Diseases Institute, Iasi, Romania; ^2^“Grigore T. Popa” University of Medicine and Pharmacy, Iasi, Romania; ^3^Nephrology Clinic, Dialysis and Renal Transplant Center, “C.I. Parhon” University Hospital, Iasi, Romania; ^4^The Academy of Romanian Scientists (AOSR), Bucharest, Romania; ^5^Department of Cardiology, Emergency University Hospital, Bucharest, Romania; ^6^“Carol Davila” University of Medicine and Pharmacy, Bucharest, Romania; ^7^The Romanian Academy of Medical Sciences, Bucharest, Romania; ^8^Department of Cardiovascular Surgery, Cardiovascular Diseases Institute, Iasi, Romania

## Abstract

**Introduction:**

Including healthcare professionals dealing with cardiovascular diseases, Heart Team is a concept/structure designed for selecting diagnostic strategies, facilitating therapeutic decisions, and improving cardiovascular outcomes in patients with complex heart pathologies, requiring input from different subspecialties and the necessity of a multidisciplinary approach. The aim of this narrative review is to search for and to summarize current evidence regarding Heart Team and to underline the future directions for the development of this concept.

**Methods:**

We searched the electronic database of PubMed, SCOPUS, and Cochrane CENTRAL for studies including Heart Team. Forty-eight studies were included, if reference was made to Heart Team structure and functionality.

**Results:**

We depicted the structure and the timeline of Heart Team, along with actual evidence-based recommendations from European Guidelines. We underlined the importance of quality of knowledge-sharing and decision-making inside the Team, analyzing bad decisions which did not reflect members' true beliefs due to “*uniformity pressure, closed mindedness, and illusion of invulnerability*.” The observation that Guidelines' indications regarding Heart Team carry a level C indication underlines the very future of this Team: randomized controlled trials proving solid benefits in an evidence-based world.

**Conclusions:**

Envisioned as a tool for optimizing the management of various complex cardiovascular pathologies, Heart Team should simplify and facilitate the activity in the cardiovascular ward. Finally, these facts should be translated into better cardiovascular outcomes and a lower psychological distress among Team participants. Despite all future changes, there must always be a constant part: the patient should remain at the very center of the Team.

## 1. Introduction: Definitions and Timeline

Composed by various healthcare professionals dealing with cardiovascular diseases, the Heart Team is a concept and a structure designed for selecting diagnostic strategies, facilitating therapeutic decisions, and improving cardiovascular outcomes in patients with complex heart pathologies [1]. The very center of Heart Team is the patient him/herself, as he/she has the last word in deciding which therapy should be performed [2].

Development of treatment strategies and options along with the proliferating amount of scientific information from various clinical trials and the need of input from different subspecialties outline the necessity of a multidisciplinary approach [3]. Moreover, studies in which revascularization techniques were underused were associated with a significantly increased mortality rate during the follow-up [4]. Therapeutical decision can be straightforward in patients with less complex coronary artery disease, but in those with complex issues/comorbidities such as diabetes mellitus (with microvascular and macrovascular complications), chronic kidney disease, advanced heart failure, and advanced age, it becomes increasingly nuanced [5].

In the early 80s, two important studies demonstrated the benefits of surgery versus medical treatment in stable coronary artery disease. Both studies used Heart Teams (including a cardiac surgeon and a clinical cardiologist) to select patients for eligibility in randomization [6, 7].

In the year 2000, the results of the EAST registry suggested that the selection of revascularization treatment after discussion with a cardiologist, cardiac surgeon, and the patient provided better outcomes (e.g., three-year survival) in comparison with randomization [8].

The introduction of SYNTAX I and II scores [9], SYNTAX functional score [10] (based on coronary flow reserve), and Clinical SYNTAX score pointed out that it is a difficult task for a professional to decide/recommend a therapy on him/her individual own [11]. Moreover, recent studies suggested that intraobserver and interobserver variability when interpreting a coronary angiogram often results in inappropriate revascularization strategies [12–14]. A Heart Team minimizes the errors and facilitates evidence-based decisions in such situations.

Since 2010, there are specific solid indications (class I recommendation) to discuss and treat complex coronary patients through a Heart Team solution [15]. Nowadays, each European Society of Cardiology (ESC) Guideline has dedicated recommendations to evaluate complex situations inside a Heart Team.

The aim of this paper is to describe the timeline of the Heart Team's structure and discuss the functionality of this team through the lens of the quality of members' interactions. Also, we intend to summarize current evidence regarding Heart Team and to underline the future directions for the development of this concept. To note, we further evaluate and present information from all recent ESC Guidelines with respect to the Heart Team indications (with classes of recommendations and levels of evidence).

## 2. Methods

We searched the electronic database of PubMed, SCOPUS, and the Register of Controlled Trials (Cochrane CENTRAL) from its earliest date until May 2019 for papers that evaluated Heart Team structure and functionality. The terms used for searching were “*heart team*,” “*clinical decision team*,” “*multidisciplinary decision making*,” “*information-sharing*,” “*groupthink*,” “*coronary heart team*,” and “*valvular heart team*.” The reference sections of the relevant articles were manually searched for additional articles (for example, we looked for psychological studies referring to the functionality of a Team from references [16]). Randomized controlled trials, observational studies, including case-control studies, prospective or retrospective cohort studies, reviews, meta-analyses were included if reference was made to the Heart Team. Case reports were excluded.

Studies were selected by two independent reviewers by screening the title and abstract. Duplicates were excluded both manually and through Reference Manager software. Of these, only 48 met the inclusion criteria. For the selected studies, we reviewed the full-text article and additional relevant publications were added after screening the reference section.

## 3. The Structure and Dynamics of the Heart Team

The Heart Team structure is obviously defined by its functionality and objectives.

A complex coronary disease situation requires both a cardiac surgeon and an interventional cardiologist [17]. In addition, a clinician cardiologist and a cardiac imagist are often required for gathering complete information and expertise in special cases [18, 19].

Other medical specialists can join the Team meetings depending on the complexity of the case (e.g., radiologist). An anesthesiologist can assess the surgical risk for a patient who may undergo CABG and give insight about the safety of general anaesthesia. Moreover, a nephrologist specialist could help with those situations in which dialysis is contemplated. A psychologist, physical therapist, and geriatrician could be involved in establishing a therapeutic strategy for severe heart failure patients [20]. As depression and anxiety have been found to be highly prevalent in cardiovascular patients, collaborative care and associated integrated care programs have been developed to manage mental health conditions in patients with cardiovascular disease. Such approaches to mental health assessment can be requested and included in Heart Team protocols [21].

The Heart Team not only meets for coronary heart disease, but also for complex valvular pathologies (to decide on treatment indication and how to replace the valve: surgical, hybrid, or interventional approaches) or congenital cardiac pathologies that may benefit from modern therapies (be it minimal intervention invasive, either surgical or hybrid). Likewise, the pathology of the aorta (complex aneurysms or dissection of the aorta) requires the Team discussions in order to choose the treatment modality and to manage the possible complications [22].

Residents and/or schooled research nurses could gather the necessary data to interpret, and share the prepared score assessments on a plenary screen [1]. Through this way, definition, typing, or calculation errors can be avoided by a feedback of the whole team [1]. Involvement of patients' families and friends in the Heart Team can increase patient satisfaction [23]. Moreover, a specialized Heart Team may ask for additional testing to help determine the most appropriate treatment option for each patient.

In a 2016 survey among involved physicians, the most important characteristics of a Heart Team were as follows: “*collaborative*,” “*multidisciplinary*,” “*beneficial*,” “*necessary*,” and “*positive*” [24]. To note, at the question “*what are the biggest barriers that prevent your Heart Team from functioning optimally?*,” the professionals answered that the sense of superiority to other staff opinions, the pressure from hospital to send more patients to surgery, and the financial reimbursement for the time and resources involved could be the most important drawbacks to a real functionality.

A prospective cohort study of 3045 CABG patients treated in 16 hospitals showed that a “*supportive group culture*” in hospitals was significantly correlated with higher patients' physical and mental health scores as determined by SF-36 questionnaires 6 months postsurgery [25].

The way information is made available through the Heart Team differs from unit to unit and a multidisciplinary team proforma was filled in prior to the meeting in a tertiary referral coronary/cardiac surgery unit from Wolverhampton—United Kingdom, so the important data are required to be at hand during the meeting [17], while other units prefer to manage all the documents from a dedicated electronic archive [26]. All the data are collected and put together prior to the meeting by nurses, resident physicians, or a multidisciplinary team coordinator [17].

Recent research studies on the efficiency of Heart Team gathering identified three main elements influencing decision-making: communicating up-to-date knowledge between various specialist and between doctors and patients; making time for discussions and listening all the inquires; and reaching an agreement on which revascularization strategy will be performed with patient preferences prioritized. Given the implementation and the purposes of this multidisciplinary approach, considerations of Heart Team as a form of standard medical care are being taken [27, 28].

## 4. Evidence-Based Heart Team

Adapted from the 2010 ESC/EACTS Guidelines [12], the first recommendations regarding Heart Team functionality are available in the 2013 ESC Guidelines on the management of stable coronary artery disease [29] (see Table 1). Since then, another 6 ESC Guidelines implemented Heart Team recommendations (most of them being class I indication and C level of evidence) [30–35].

The reader should be aware that most of the Guideline recommendations are level of evidence C, a level of expert consensus requiring further solid studies for reinforcing evidence.

An ESC Review dealing with the structure of the Heart Team [1] identified five directions for new studies: (a) exploring the reproducibility of the Heart Team by presenting treatment decision of specific cases to different medical groups (teams in different regions or teams with different structures); (b) evaluating intraobserver variability to treatment recommendation (comparing an initial specialist evaluation to a reassessment by the Heart Team); (c) RCTs evaluating patients' outcomes either through a Heart Team decision or according to the original recommendations by the surgeon or cardiologist; (d) “*before-and-after studies*” comparing treatment decisions and outcomes before and after implementation of the Heart Team; and (e) comparison of treatment decisions and outcomes of different centres with and without Heart Team evaluation.

## 5. Team Interactions *versus* Team Outcomes

It is obvious that the main objective of Heart Team's planning was to optimize treatment decisions and improve cardiovascular patients' outcomes. Moreover, the management of resources was another important target of the Heart Team. However, there are attention signals that all these concepts function smoothly only in theory [16], prior biases and the manager culture in hospitals being two major drawbacks [36]. Therefore, the next step to improving Heart Team functionality and quality of decisions should be implementing effective standards which minimize errors.

Currently, there are no dedicated standards regarding the type of discussions and the ways to solve the lack of consensus [37]. Recent psychological studies revealed that knowing others' preferences (especially leader's decisions) degrades the quality of group final recommendations [38]. The decision-making process seems to be considered an outcome rather than a process [39].

Various research studies found that politics and medical groups yielded bad decisions which did not reflect team members' true beliefs due to “*uniformity pressure, closed mindedness*, *and illusion of invulnerability*” [40]. One of the most important “*myths*” related to Heart Team functionality is that if team members have concerns regarding treatment, they will also voice them [16]. Unfortunately, lack of speaking up with ideas poses a serious bias to the final recommendation [41].

Team leaders should be opened and invite/appreciate dissent helping members to express their ideas, opinions, and concerns [42]. Also, one of the solutions to this issue is that team leaders should be responsible not only for an outcome but also for the decision-making process [16]. Moreover, leadership style has a tremendous impact on decision quality, “*groups with directive (rather than participative) leaders more often make decisions consonant with leader's initial information*” [16, 43]. Discouraging others through leader's point of view seems to be an ineffective way of establishing a Heart Team final decision [44].

However, biases could also be found in Heart Team members: advocacy-based process (in which members become motivated to win their point by arguing and proving other perspectives are wrong) [45] and “*anchoring bias*” (once a doctor has a decision, it becomes difficult to recognize it was wrong) [16]. Another important element is that decisions should be taken through discussion rather than voting (the majority rule forces the minority to compromise, which can mask deep disagreement) [46]. A psychologically safe atmosphere inside the Team in addition to a “*gentle art of asking instead of telling*” proves to be the key element to a real useful decision [47].

## 6. The Future of Heart Team: Where Are We Heading to?

“*Underutilization, overutilization, and inappropriate use*” of advanced and expensive cardiovascular therapies should be directly addressed by the Heart Teams [1].

As choices and treatments are now more complex, “*the future of cardiology jobs may rest in Heart Teams.”* [3] Moreover, as minimally invasive cardiac surgery develops and robotically assisted coronary bypass surgery and hybrid coronary revascularization procedures are emerging, we are witnessing a continuous changing in the management of various valvulopathies and ischaemic coronary disease [48].

The above observation that all Guidelines' indications regarding Heart Team activation carry a Level C indication underlines the very future and the needs of this new Team: randomized controlled trials proving solid benefits in an evidence-based world [49]. It looks obvious that a final resolution yielded through a discussion gathering information from more specialists is more accurate and generates better cardiovascular outcomes, but until proofs, it is just a supposition.

Presently, even if the class of recommendation for gathering the Heart Team is I in various clinical situations (see Table 1), not every hospital has local protocols for activating it [50]. Moreover, Heart Team seems to be an “*ideal platform for recruitment into trials of PCI vs CABG”* as SYNTAX teams proved to be [51]. In addition, not all the hospitals detain on-site cardiac surgery, which makes the decisions biased toward PCI [52] (a situation almost similar to the contrary situation, as surgeons show a slight bias toward CABG) [53, 54].

Two important recent studies already revealed that, in some settings, Heart Team decisions proved to be highly reproducible and with good outcomes [26, 55]. Different study designs have been suggested to further evaluate the Heart Team concept: taking the same cases into consideration to different Heart Teams and comparing cardiovascular outcomes and treatment recommendations before and after Heart Team implementation [1, 26].

At the same time, there are contrary opinions regarding the utility of the Heart Teams: “*as it stands today, “Heart Team” is more of a fictional euphemism, a kind of 'Platonic Illusion' rather than a pragmatic reality.*” [56] The future studies though should provide solid financial arguments and better survival rates in order to both sustain its complex decisions and justify its financial support. There is a need for new studies investigating the significance of delays in the decision-making process of Heart Team as well as financial aspects regarding its gatherings (healthcare providers reimburse Heart Teams for its decisions—in which inappropriate coronary revascularisations are lowered and outcomes are improved) [1].

Moreover, there is a need for web-based algorithms to offer interactive details (for both patients and professionals) on different therapies and strategies (similar to those from SYNTAX score II) [57]. An interactive computer program proved to be more effective than standard genetic counseling in educating breast cancer patients and reducing anxiety as well as increasing accurate risk perception [58]. Besides Heart Team management, a computer software dedicated for cardiovascular patients will better implement its evidence-based recommendations. As other research studies demonstrated, a further step should be considered when the patient himself/herself will attend their own discussion when Heart Team is functioning [17].

## 7. Conclusions

Envisioned as a tool for optimizing the management of various complex cardiovascular pathologies (coronary diseases, valvular pathology, and congenital diseases), Heart Team should therefore simplify and facilitate the activity in the cardiovascular ward dealing with complex situations. Future improvement of its functionality (members' interactions, the way knowledge is transmitted throughout the Team, feedback algorithms, and patient involvement in the decisional process) and subsequent randomized controlled trials would increase the Team's importance and implication in the management of complex cases. Finally, these facts should be translated into better cardiovascular outcomes and a lower psychological distress among Team participants. Despite all future changes, there must always be a constant part in each Heart Team: the patient should remain at the very center of the Team.

## Figures and Tables

**Table 1:** Heart Team approach recommendations through the ESC Guidelines referring to coronary artery diseases.

*2013 ESC Guidelines on the management of stable coronary artery disease*		LOE
Indications for revascularization of stable CAD patients on optimal medical therapy (adapted from ESC/EACTS 2010 Guidelines)	A Heart Team approach to revascularization is recommended in patients with unprotected LM, 2-3 vessel disease, diabetes, or comorbidities	IC

*2015 ESC Guidelines for the management of acute coronary syndromes in patients presenting without persistent ST-segment elevation*		
Recommendations for perioperative management of antiplatelet therapy in non-ST-elevation acute coronary syndrome patients requiring CABG	It is recommended that the Heart Team estimates the individual bleeding and ischaemic risks and guides the timing of CABG as well as management of DAPT	IC
Recommendations for invasive coronary angiography and revascularization in non-ST-elevation acute coronary syndrome	In patients with multivessel CAD, it is recommended to base the revascularization strategy (e.g., ad hoc culprit-lesion PCI, multivessel PCI, and CABG) on the clinical status and comorbidities as well as the disease severity (including distribution, angiographic lesion characteristics, and SYNTAX score), according to the local Heart Team protocol	IC
Recommendations for the management of patients with acute heart failure in the setting of non-ST-elevation acute coronary syndromes	It is recommended that patients with mechanical complications of NSTEACS are immediately discussed by the Heart Team	IC

*2016 ESC Guidelines for the management of atrial fibrillation developed in collaboration with EACTS*		
Recommendations for catheter ablation of atrial fibrillation and atrial fibrillation surgery	Minimally invasive surgery with epicardial pulmonary vein isolation should be considered in patients with symptomatic AF when catheter ablation has failed. Decisions on such patients should be supported by an AF Heart Team	IIaB
	Maze surgery, possibly via a minimally invasive approach, performed by an adequately trained operator in an experienced center, should be considered by an AF Heart Team as a treatment option for patients with symptomatic refractory persistent AF or postablation AF to improve symptoms	IIaC

*2017 ESC focused update on dual antiplatelet therapy in coronary artery disease developed in collaboration with EACTS*		
DAPT in patients treated with cardiac surgery with stable or unstable CAD	It is recommended that the Heart Team estimates the individual bleeding and ischaemic risks and guides the timing of CABG as well as the antithrombotic management	IC

*2017 ESC Guidelines for the management of acute myocardial infarction in patients presenting with ST-segment elevation*		
Recommendations for the management of cardiogenic shock in ST-elevation myocardial infarction	It is indicated that mechanical complications are treated as early as possible after discussion by the Heart Team	IC

*2017 ESC/EACTS Guidelines for the management of valvular heart disease*		
Indications for surgery in (A) severe aortic regurgitation and (B) aortic root disease (irrespective of the severity of aortic regurgitation)	Heart Team discussion is recommended in selected patients in whom aortic valve repair may be a feasible alternative to valve replacement	IC
Indications for intervention in aortic stenosis and recommendations for the choice of intervention mode	Aortic valve interventions should only be performed in centres with both departments of cardiology and cardiac surgery on-site and with structured collaboration between the two, including a Heart Team (heart valve centres)	IC
	TAVI is recommended in patients who are not suitable for SAVR as assessed by the Heart Team	IB
	In patients who are at increased surgical risk (STS or EuroSCORE II>_4% or logistic EuroSCORE I>_10% or other risk factors not included in these scores such as frailty, porcelain aorta, and sequelae of chest radiation), the decision between SAVR and TAVI should be made by the Heart Team according to the individual patient characteristics, with TAVI being favoured in elderly patients suitable for transfemoral access	IB
	SAVR should be considered in patients with moderate aortic stenosis undergoing CABG or surgery of the ascending aorta or of another valve after Heart Team decision	IIaC
Indications for intervention in severe primary mitral regurgitation	Percutaneous edge-to-edge procedure may be considered in patients with symptomatic severe primary mitral regurgitation who fulfil the echocardiographic criteria of eligibility and are judged inoperable or at high surgical risk by the Heart Team, avoiding futility	IIbC
Indications for mitral valve intervention in chronic secondary mitral regurgitation	In patients with severe secondary mitral regurgitation and LVEF <30% who remain symptomatic despite optimal medical management (including CRT if indicated) and who have no option for revascularization, the Heart Team may consider a percutaneous edge-to-edge procedure or valve surgery after careful evaluation for a ventricular assist device or heart transplant according to individual patient characteristics	IIbC
Management of prosthetic valve dysfunction—haemolysis and paravalvular leak	Transcatheter closure may be considered for paravalvular leaks with clinically significant regurgitation in surgical high-risk patients (Heart Team decision).	IIbC
Management of prosthetic valve dysfunction—bioprosthetic failure	Transcatheter valve-in-valve implantation in the aortic position should be considered by the Heart Team depending on the risk of reoperation and the type and size of prosthesis	IIaC

*2018 ESC/EACTS Guidelines on myocardial revascularization*		
Recommendations for decision-making and patient information in the elective setting	It is recommended that institutional protocols are developed by the Heart Team to implement the appropriate revascularization strategy in accordance with current guidelines	IC
Recommendations on revascularization in patients with chronic heart failure and systolic left ventricular dysfunction (ejection fraction < 35%)	In patients with three-vessel disease, PCI should be considered based on the evaluation by the Heart Team of the patient's coronary anatomy, the expected completeness of revascularization, diabetes status, and comorbidities	IIaC
Recommendations for the management of patients with cardiogenic shock	In cases of haemodynamic instability, emergency surgical or catheter-based repair of mechanical complications of ACS is indicated, as decided by the Heart Team	IC
Recommendations on repeat revascularization—early postoperative ischaemia and graft failure	It is recommended that either emergency reoperation or PCI is decided upon by ad hoc consultation in the Heart Team, based on the feasibility of revascularization, area at risk, comorbidities, and clinical status	IC
Recommendations on repeat revascularization—restenosis	In patients with recurrent episodes of diffuse in-stent restenosis, CABG should be considered by the Heart Team over a new PCI attempt	IIaC
DAPT in patients undergoing cardiac surgery	It is recommended that the Heart Team estimates the individual bleeding and ischaemic risks and guides the timing of CABG as well as the antithrombotic management	IC

CAD: coronary artery disease; LM: left main; CABG: coronary artery bypass grafting; DAPT: dual antiplatelet therapy; PCI: percutaneous coronary intervention.
